# Predicting leaf traits in wine grapes with reflectance spectroscopy

**DOI:** 10.1371/journal.pone.0336560

**Published:** 2026-05-18

**Authors:** Rachel O. Mariani, Marney E. Isaac, Kimberley A. Cathline, Gavin Robertson, Adam R. Martin

**Affiliations:** 1 Department of Physical and Environmental Sciences, University of Toronto Scarborough, Scarborough, Ontario, Canada; 2 Horticultural & Environmental Sciences Innovation Centre, Niagara College, Toronto, Ontario, Canada; University of Innsbruck, AUSTRIA

## Abstract

Estimating crop trait data is critical for predicting crop responses to environmental change, enabling more informed diagnoses of crop performance and the development of on-farm management strategies. Yet, many traditional methods for quantifying plant traits are time-consuming and resource-intensive, limiting sample sizes and study durations. In response, high-throughput phenotyping—specifically reflectance spectroscopy—has emerged as a key element of plant trait research, enabling rapid estimation of plant traits. However, little is known about whether reflectance spectroscopy can detect within-species variation in resource acquisition and plant-water traits, especially variation that exists among different cultivars or genotypes of the same crop. Using wine grapes (*V. vinifera* subsp. *vinifera*) as a focal crop, this study aimed to assess the ability of reflectance spectroscopy to quantify intraspecific variation in 12 leaf traits across 12 different cultivars from seven different varieties. We find significant variability in traits across and within cultivars, especially in gas-exchange and hydraulic traits, with cultivars varying along a resource-conservative-to-resource-acquisitive trait axis. Models based on spectral reflectance data were able to differentiate and predict this fine-scale trait variation among cultivars for seven plant traits, with a predictive power range of *R*^2^ = 0.12–0.57. Models predicting leaf chemical (i.e., carbon and nitrogen concentrations), physiological (i.e., maximum rate of light-saturated photosynthesis), and morphological traits (i.e., leaf dry matter content) were more accurate in their predictions, while models predicting leaf water status were less accurate. Our results indicate that reflectance spectroscopy can capture certain dimensions of the fine-scale trait variation that exists within genetically diverse agroecosystems, though spectroscopic estimates of intraspecific variation in leaf water status are less accurate.

## 1 Introduction

In agricultural systems, environmental change driven by deviations in temperature and precipitation patterns is exerting profound impacts on crop growth and productivity [1,2]. While observations and predictions vary widely across crop species and geographies, recent studies have reported anywhere between 7–23% declines in crop yield [1]. Such changes largely represent the impact of fluctuations in precipitation and changes in growing and non-growing season temperatures on crop phenology, ecophysiology, and nutrient uptake [3]. For example, with gradual shifts towards overall warmer winters in many of Canada’s agricultural regions, crops are expected to face reduced cold-hardiness, resulting in increased susceptibility to spring frost damage to early vegetative growth, including primary buds [4,5]. These and other complex interactions between environmental impacts and individual plants scale up to influence crop performance, yield, and quality on farms (e.g., [2–4]).

Globally, wine grapes are one of the world’s most valuable crops [2]. In the province of Ontario, Canada—the region of our study—wine is among the highest-value-added agricultural products, generating a total economic impact of $5.49 billion in 2024−25 [6]. However, the suitability of the world’s wine-growing regions is expected to shift in the coming years and decades. Rising temperatures and prolonged droughts may hinder grape and wine production, making hot and dry regions less suitable for cultivation. Meanwhile, mid-latitude wine-growing regions could face a higher risk of spring frosts and increased precipitation, which are expected to impact plant phenology with negative consequences for production [2]. Wine grapes are also vulnerable to other aspects of environmental change, such as temperature variability, precipitation fluctuations, and extreme weather events, all of which can individually or collectively influence grapevine growth and fruit quality [5,7].

Projected shifts in climate pose significant challenges in viticulture. Predictions suggest that under a 2 °C global temperature increase scenario, up to 56% of all current wine-growing regions will become unsuitable for growing due to changes in grapevine phenology and grape composition, with this number increasing to 85% with a 4 °C increase by the end of the century [2,8,9]. Thus, wine growers now consider adaptation strategies to ensure consistency in production and quantity under variable environmental conditions [2,10]. More specifically, varietal selection and cultivar turnover are potentially key climate change adaptation strategies to ensure vine resilience to changes in growing regions. Cultivar turnover relies on varietal selection, where grapevine varieties are chosen based on their tolerance to certain environmental conditions, and are planted using various combinations of scion, rootstock, and vine training systems [11]. Wine grapes exhibit significant genomic and phenotypic diversity due to years of artificial selection, resulting in cultivars with traits that confer resistance or resilience towards environmental variability.

Plant responses to environmental change drivers can be multifaceted and governed by synchronized and/ or independent leaf, root, and whole-plant stress responses [1]. One key hypothesis in ecosystem science is that inter- and intra-specific variation in leaf functional traits—the characteristics of plants that determine responses to the surrounding environment—play a role in governing plant- and ultimately ecosystem-level responses to climate change [12–14]. Leaf traits related to the exchange of CO_2_ and water, as well as a suite of other morphological, chemical, and physiological traits, are especially influenced by both environmental and genetic factors [11,15]. Generally, the literature on functional traits supports the expectation that greater functional trait variation leads to greater ecosystem resistance and/ or resilience to environmental change [16–18]. While earlier research in this area focused on characterizing the causes and consequences of trait variation across species, more recently, research suggests that higher intraspecific trait variation also confers stronger resistance and adaptation of plant communities to change [16].

Quantifying trait variation with crop species is particularly important in agroecosystems, where a limited number of plant species, varieties, or cultivars dominate and ultimately influence ecosystem functioning due to their high abundance [19]. However, many “traditional” methods for characterizing traits, especially those related to leaf-level CO_2_ and water fluxes [20,21], are often time-consuming and resource-intensive as they rely on low-throughput tools such as infrared gas analyzers or Scholander-type pressure chambers. For example, pressure-volume curves, used to generate data on plant drought tolerance traits or plant water status, can take multiple hours to complete [16,20,21]. Gas exchange systems used to estimate physiological traits such as maximum photosynthetic capacity, stomatal conductance, or water-use efficiency are likewise time-consuming [22]. In response to such challenges associated with plant trait data collection, high-throughput phenotyping (HTP) has emerged as vital in plant trait research. Compared to “traditional” data collection methods, HTP tools can rapidly quantify several plant traits in a field setting [23].

At the ecosystem level, remote sensing is a key component of HTP. At the plant- or leaf-level, proximal reflectance spectroscopy is now more commonly being used to assess variation in traits across plant species [24,25], particularly those associated with the Leaf Economics Spectrum (LES), such as leaf mass per area (LMA), light-saturated photosynthetic capacity (*A*_max_), and leaf nitrogen concentrations (N). Earlier research in this area primarily focused on employing reflectance spectroscopy to quantify trait variation across species, especially among plants of different functional types with diverse evolutionary histories [26]. Here, reflectance spectroscopy has proven effective in predicting trait variation and values, especially for leaf morphological and physiological traits, suggesting that this technique is suitable for rapid trait assessment in natural and managed ecosystems.

Less is known about the ability of reflectance spectroscopy to quantify finer-scale trait variation that exists within species [18,25,26]. For crops in agroecosystems, both genetic [18] and environmental factors [11] constrain or govern intraspecific trait variation. Characterizing differences in trait expression across cultivars of the same species is especially important in wine grapes, where individual fields and farms often cultivate multiple varieties (e.g., Cabernet sauvignon, Pinot noir) and cultivars of the same varieties (e.g., Pinot noir clones 89 and 828) [7]. A number of studies have quantified the considerable variation that exists among traits across wine grape varieties growing on the same vineyards [5,27–29]; yet, fewer studies have detailed the trait variation that exists among varieties and cultivars growing under otherwise similar environmental conditions. Similarly, research, including our own, has shown that reflectance spectroscopy predicts photosynthetic C assimilation traits, at least across wine grape varieties that differ widely in their domestication histories and, in turn, trait expression [30]. However, to our knowledge, no studies have tested whether or not reflectance spectroscopy can predict the (presumably) smaller differences in trait expression that exist within varieties, specifically among closely related cultivars.

Additionally, uncertainty remains regarding which specific traits can be predicted by spectroscopic techniques. Specifically, while numerous studies have employed reflectance spectroscopy to characterize plant traits related to leaf-level carbon gain, morphology, and stoichiometry—including our previous studies on wine grapes [30]—fewer have used this HTP approach to assess within-species variation in leaf water relations traits [26,31,32]. Leaf physiological traits, including transpiration (*E*), maximum photosynthetic capacity (*A*_max_), and stomatal conductance (*g*_s_), are central to understanding leaf- and plant water-use efficiency (WUE)—a major focus of this research—as high WUE is associated with enhanced crop performance under water-limited conditions [33,34].

More specifically, to the best of our knowledge, and despite their ecological and agronomic importance, limited studies have evaluated whether or not reflectance spectroscopy can predict leaf-level isotopic signatures associated with intrinsic water-use efficiency (δ ^13^C) or predawn water potential (Ψ_pd_): two traits that are critical indicators of plant water relations and water-use strategies [35,36]. Both traits are key in assessing crop drought tolerance by providing measures of stomatal behaviour and plant water status, which regulate carbon assimilation, transpiration, and water-use efficiency under periods of environmental stress. In particular, δ ^13^C reflects long-term stomatal conductance, serving as a proxy to determine water-use efficiency over time [36]. Comparatively, Ψ_pd_ provides an estimate of baseline plant water status and informs an understanding of water limitation experienced by individual plants [35]. Using reflectance spectroscopy to estimate δ ^13^C and Ψ_pd_ could therefore be key to continuous monitoring of plant- and farm-level plant-water relations across growing seasons.

This study aims to characterize variation in leaf traits associated with plant-water relations and aboveground resource acquisition across multiple wine grape varieties and cultivars growing on the same farm, to assess the following: 1) Do wine grape varieties and cultivars differ in their leaf traits, specifically in their leaf morphological, chemical, physiological, and water-use traits? If so, 2) Can reflectance spectroscopy quantify this trait variation? We hypothesize that wine grape varieties and cultivars will exhibit significant differences in leaf traits, reflecting genetic variation (i.e., among cultivars), especially in physiological and water-use traits, which tend to vary more widely across varieties than morphological and/ or chemical traits [17,18,33,37,38]. Additionally, we hypothesize that more variable leaf traits, namely those associated with leaf physiological functioning, will be more accurately predicted by reflectance spectroscopy-based models than less variable morphological and chemical traits.

## 2 Materials and methods

### 2.1 Study design and trait collection

This study was conducted at the Niagara College Teaching Vineyard in Niagara-on-the-Lake, Ontario (43.146237 °N, −79.156810 °W). The study site is an operational teaching vineyard containing multiple wine grape cultivars (*V. vinifera* subsp. *vinifera*) distributed across the 16.2-ha farm in rows separated by either cover crops or unmanaged grasses. The soil at the vineyard is a mineral-rich mix of sand, gravel, loam, and red clay, creating an ideal substrate for wine grape production. At the site, 12 different wine grape cultivars belonging to seven different varieties of both red and white grapes are present, including Cabernet franc clones 327 and 314 (CF327 and CF314), Cabernet sauvignon clones 29 and 412 (CS29 and CS412), Merlot clones 384 and 181 (M348 and M181), Pinot noir clones 89 and 828 (PN89 and PN828), Riesling clones 23 and 171 (R23 and R171), Sauvignon blanc clone 906 (SB906), and Viognier clone 642 (V642). All cultivars are grown on a Selection Oppenheim (SO4) rootstock except Viognier 642, which is grown on the Millardet et de Grasset (101−14) rootstock. The vine training system is primarily a vertical shoot positioning system, most commonly used for varieties that thrive under improved sunlight exposure. The vineyard is not irrigated but tile-drained.

For each of the 12 cultivars, five vines in three different planting rows equally spaced along the northwest side of the vineyard were selected for functional trait characterization. Specifically, a total of *n* = 178 leaves were sampled from *n* = 15 vines per cultivar equally across *n* = 12 cultivars at the vineyard. On each vine, we selected one fully expanded, west-facing upper canopy leaf for reflectance spectroscopy and trait collection, such that all selected leaves were approximately the same size and free from any visible signs of damage. All upper canopy leaves were between 1 and 1.5 m in height at the time of sampling.

For each sample leaf, two main sets of in-field measurements were collected. First, physiological gas exchange data were collected using an LI-6800 portable photosynthesis system affixed with a 6800−03 large light source (LICOR Biosciences, Lincoln, Nebraska, USA). Specifically, for each leaf in our dataset the LI-6800 was used to measure stomatal conductance (*g*_s_; mol m^-2^ s^-1^), light-saturated photosynthetic capacity (*A*_max_; *μ*mol CO_2_ m^-2^ s^-1^), and transpiration (*E*; mmol H_2_O m^-2^ s^-1^), under the following leaf chamber conditions: CO_2_ concentrations of 420 ppm, saturating light levels of 1,500 *μ*mol of photosynthetically active radiation (PAR) m^-2^ s^-1^ (with irradiance peaks in blue [453 nm], green [523 nm], and red [660 nm] wavebands), relative humidity (RH) of 50%, leaf vapour pressure deficits (VPD) of 1.7 MPa, and leaf temperatures of 25 °C (Macklin, et al., 2022). All physiological trait measurements were taken between 7:00 and 13:00 to ensure the leaves were not approaching stomatal closure. To ensure the leaves acclimatized to the leaf chamber conditions, we waited approximately 5 minutes after securing the sensor head to the leaf before taking measurements, and measurements were recorded only once leaves had stable *A*_max_ readings. Additionally, to ensure stomata were not closed during measurements, no measurements were taken if *g*_s_ fell below 0.06 mol^-2^ s^-1^. Physiological traits measured in the field were used to determine intrinsic water-use efficiency (WUE_intr_; *μ*mol CO_2_ mol^-^¹ H_2_O) calculated as *A*_max_/*g*_s_, and instantaneous water-use efficiency (WUE_inst_; *μ*mol CO_2_ mmol^-^¹ H_2_O) calculated as *A*_max_/*E*.

Following gas-exchange measurements, we collected spectral reflectance data for each leaf using an SVC HR-1024i handheld spectroradiometer equipped with an LC-RP Pro leaf clip that included a calibrated internal light source (Spectra Vista Corporation, Poughkeepsie, New York, USA). The SVC HR1024i is a full-range spectroradiometer covering 350–2500 nm, with a spectral resolution of ≤ 3.5 nm (350–1000 nm), ≤ 9.5 nm (1000–1800 nm), and ≤ 6.5 nm (1800–2500 nm). Reflectance spectra were collected on the adaxial side of the leaves with an integration time of 2 s, and reference spectra were taken on a white Spectralon standard before each measurement. All reflectance spectroscopy measurements were taken immediately after predawn water potential measurements.

To determine pre-dawn water potential, all leaves sampled for physiological measurements and reflectance spectroscopy were removed from their vines before sunrise (between 03:30 and 05:00) the morning following gas exchange measurements (i.e., generally within 12–14 hours). Leaves were stored in darkness while maintaining leaf hydration by wrapping each sample in a saturated paper towel, sealing it in a polyethylene bag, and storing it in a cool, insulated bag that blocked all sunlight until they were successfully transported. Predawn water potential measurements were executed using a Portable Plant Water Potential Console (Soil Moisture Equipment Corp., Goleta, CA., USA). For each leaf, a clean blade was used to slice through the petiole across the horizontal axis, and the leaf was inserted into the chamber with a microscope camera aimed at the cut petiole to detect when sap was first exuded, at which point Ψ_pd_ was recorded.

Following measurements of Ψ_pd_, all leaves were transported to the University of Toronto, Scarborough, Canada, for measurement of morphological and chemical traits. First, all leaves were weighed for leaf fresh mass (g) and scanned for fresh leaf area (cm^2^) using an LI-3100C leaf area meter (LICOR Biosciences, Lincoln, NE, USA). Subsequently, all leaves were dried at 60 °C and reweighed to determine dry mass (g), which was then used to calculate leaf dry matter content (LDMC) as mg of dry mass/ g of fresh mass. Following these steps, leaves were ground into fine powder using a MM40 Retsch ball mill (Retsch Ltd., Hann, Germany), and leaf N and C concentrations were determined on 0.102–0.156 g of leaf tissue using a LECO CN 628 elemental analyzer (LECO Instruments, Ontario, Canada). Then, LMA was calculated as leaf dry mass (g)/ leaf area (m^2^), and LDMC (mg g^-1^) was calculated as leaf dry mass (mg)/ leaf saturated mass (g). Finally, we assessed δ ^13^C on ~1 mg of powdered leaf tissue using a Picarro G2131-i isotope and gas concentration analyser (Picarro Inc., Santa Clara, CA, USA).

### 2.2 Statistical analyses

We used R statistical software v. 4.4.3 (R Foundations for Statistical Computing, Vienna, Austria) for all data analysis. First, we calculated the summary statistics for all leaf traits and assessed normality using both a Shapiro–Wilk test and visual inspection with histograms and quantile-quantile plots, performed using the base R “stats” package. Then, variance component estimation was performed using the “nlme” R package [39,40] using a nested model approach with the following components: origin (originating from a warm or cool growing region), red/white varieties, cultivar, and planting row. We then performed an analysis of variance (ANOVA) with a Tukey’s Honestly Significant Difference post-hoc test to assess significant trait differences among cultivars.

Following these steps, we then performed analysis designed to test whether or not reflectance spectroscopy predicts leaf trait values using chemometrics, specifically partial least squares regression analysis (PLSR), following published protocols [24]. To do so, we first detected and removed outliers using the interquartile range (IQR) method with a factor of 2, as this approach is used for data with moderate to low variability [41]. Outliers were removed for the following leaf traits: leaf C (*n* = 1 outlier removed), δ ^13^C (*n* = 1 outlier removed), leaf area (*n* = 7 outliers removed), LDMC (*n* = 7 outliers removed), LMA (*n* = 7 outliers removed), and WUE_inst_ (*n* = 12 outliers removed). Only single trait values, and not entire leaves, were removed from the data set to maintain the largest possible sample sizes for each PLSR model (as per [24]).

We then applied the methodology outlined in [42] and [30] to assess whether reflectance spectroscopy predicts intraspecific trait variation using PLSR models. All PLSR models incorporated reflectance data across the 400–2400 nm wavelength range and were designed to predict raw and/ or log-transformed trait values (as per results from our normality tests). For each PLSR model, trait data were divided into a calibration dataset (80% of the data) and a validation set (the remaining 20%) using the “pls” and “spectrolab” R packages [43, 44]. Since we are especially interested in evaluating whether reflectance spectra can predict the smaller trait variation likely present across grape cultivars, we conducted our PLSR model analysis using two data-splitting approaches. First, we split the dataset by cultivar identity, ensuring that the calibration and validation sets contained approximately equal representation of trait and spectral data for each of the 12 cultivars. Second, we performed a fully randomized split, allowing the proportion of data from each cultivar to vary randomly.

For each calibration dataset, we then used the ‘find_optimal_components’ function in the ‘spectratrait’ R package to identify the optimal number of components used in each PLSR model [45], in conjunction with the minimum calculated prediction residual sum of squares (PRESS) statistic. Then, for each trait calibration dataset, PLSR models were fitted using a leave-one-out (LOO) cross-validation procedure (where the number of iterations is equal to *n*_*cal*_-1), implemented using the ‘plsr’ function in the ‘pls’ R package [44]. Model performance was evaluated using the validation datasets as external test datasets, by comparing predicted values vs. observed values (again, in the validation data subset only). For each PLSR model fitted to the validation dataset, we used the coefficient of determination (*R*^2^), root mean squared error of prediction (RMSE), and percent RMSE (%RMSE) as the primary model evaluation criteria. Then, we analysed the regression coefficients and variable importance in projection (VIP) scores to identify which spectral regions significantly contributed to trait prediction. Additionally, we conducted a jackknife permutation analysis implemented in the “plsr” function in the “pls” R package [44] to assess model uncertainty. The jackknife coefficient estimates were then compared with those from the full model, and we calculated the mean predicted values along with 95% confidence and prediction intervals for each trait in the validation dataset. Lastly, we used a Pearson correlated test to evaluate the relationship between the prediction *R*^2^ values from PLSR models and the extent of trait variation (as indicated by the CV for each trait).

## 3 Results

### 3.1 Trait variation across wine grape varieties and cultivars

**Fig 1:**
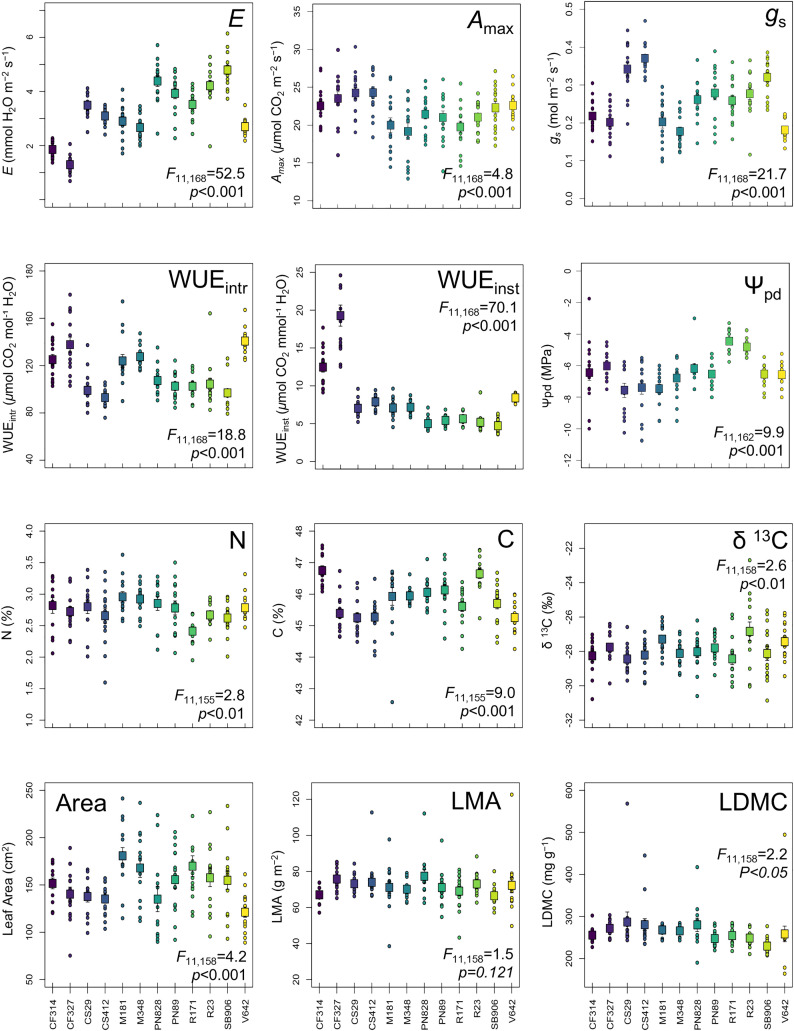
Trait variation of 178 individual plants, covering 12 cultivars of wine grapes. Individual points (circles) represent data for individual plants, the boxes represent mean values per cultivar, and the bars represent standard errors.

Descriptive statistics related to trait variation across our entire dataset are presented in Table 1, alongside variance components attributed to variety origin, red/ white grape cultivars, cultivar identity, and planting row. Almost all traits evaluated in our analysis varied significantly across cultivars (ANOVA *p* < 0.01 in all cases) except for LMA (ANOVA *p* = 0.121; Fig 1). Leaf physiological and water-use traits varied most widely across cultivars, as indicated the largest coefficients of variation (CV). More specifically, plant water-use traits including *E* varied from 0.68–6.14 mmol H_2_O m^-2^ s^-1^ (CV = 34.8%), *g*_s_ ranged from 0.1–0.47 mol m^-2^ s^-1^ (CV = 31.0%), and both WUE_inst_ and WUE_intr_ varied widely from 3.6–29.0 *μ*mol CO_2_ mmol^-1^ H_2_O (CV = 33.98%), and 44.90–174.94 *μ*mol CO_2_ mol^-1^ H_2_O (CV = 29.32%), respectively (Fig 1). Comparatively, the leaf chemical and morphological traits exhibited lower CV values. Specifically, traits expressing the lowest extent of variation across our dataset included leaf C (range = 42.57–47.54%, CV = 1.7%) and δ ^13^C (range = −30.86 to −22.66 ‰, CV = 4.4; Table 1. Generally, physiological traits related to leaf-level water fluxes, namely *E*, *g*_s_, WUE_intr_, WUE_inst_, and Ψ_pd_, varied more widely than morphological and chemical traits, with variance partitioning further indicating that these traits are especially variable/constrained across cultivars (Table 1).

**Table 1:** Descriptive statistics and variance partitioning for 12 leaf traits measured across 12 wine grape cultivars (*n* = 178).

	Descriptive Statistics									Variance Component Estimation				
Trait Group	Trait	SW	Med	MAD	$\overset{―}{X}$	SD	Min	Max	CV	Origin	R/W	Clone	Row	Unex
Physiological	*E*	0.991	–	–	3.24	1.13	0.68	6.14	34.83	0.42	<0.001	0.38	0.08	0.13
	*A*_max_	0.988	–	–	21.83	3.35	12.95	30.35	15.35	0.27	<0.001	<0.001	0.19	0.53
	*g*_s_	0.983	–	–	0.26	0.08	0.10	0.47	30.98	<0.001	<0.001	0.54	0.15	0.31
Chemical	N	0.993	–	–	2.75	0.35	1.60	3.63	12.66	<0.001	0.12	<0.001	0.05	0.83
	C	0.984	–	–	45.81	0.78	42.57	47.54	1.70	0.08	<0.001	0.32	<0.001	0.61
Water relations	δ ^13^C	0.983*	–	–	−27.88	1.22	−30.86	−22.69	4.37	<0.001	<0.001	<0.001	0.08	0.84
	Ψ_pd_	0.978*	–	–	−5.88	1.47	−10.25	−1.25	24.95	<0.001	0.18	0.21	0.07	0.55
	WUE_intr_	0.986	85.51	25.07	–	–	44.90	174.94	29.32	<0.001	<0.001	0.52	0.15	0.33
	WUE_inst_	0.925**	6.70	2.28	–	–	3.59	28.98	33.98	0.59	<0.001	0.29	0.04	0.08
Morphological	Area	0.994	150.74	34.30	–	–	75.31	289.37	22.76	0.15	<0.001	0.09	<0.001	0.77
	LDMC	0.811**	255.69	21.98	–	–	163.95	568.35	8.60	<0.001	0.09	0.05	<0.001	0.86
	LMA	0.905**	70.51	6.75	–	–	38.53	122.61	9.57	<0.001	<0.001	0.04	<0.00	0.96

*E*: mmol H_2_O m^-2^ s^-1^; *A*_max:_ *μ*mol CO_2_ m^-2^ s^-1^; *g*_s:_ mol m^-2^ s^-1^; WUE_inst_: *μ*mol CO_2_ mmol^-1^ H_2_O; WUE_intr_: *μ*mol CO_2_ mol^-1^ H_2_O; N: % dry mass; C: % dry mass; δ ^13^C: ‰; Ψ_pd_: MPa; Area cm^2^; LDMC: mg g^-1^; LMA: g m^-2^. *: *p* < 0.05; **: *p* < 0.001. Med: median; MAD: median absolute deviation; $\overset{―}{X}$: mean; SD: standard deviation; CV: coefficient of variation; R/W: red or white; Unex: unexplained.

Specifically, for traits related to plant water relations, namely *E* and WUE_inst_, the largest proportions of the variability in the trait data were explained by cultivar origin (0.42 and 0.59, respectively). Otherwise, cultivar identity explained the largest proportion of variation in *g*_*s*_ (0.54), Ψ_pd_ (0.21), WUE_intr_ (0.52), leaf C (0.32), and WUE_inst_ (0.29) (Table 1). Cultivar identity was the second-most important source of trait variation; however, a majority of the traits expressed a significant amount of ‘unexplained variance’, meaning a large portion of the variability of these traits that was not captured in our analysis or by any of our explanatory factors analyzed here. This suggests that trait values emerge from the interaction between cultivar identity and local environmental conditions; in our case, the high unexplained variance may reflect conditions associated with unmeasured soil conditions, water availability, or other environmental parameters.

Across cultivars gas exchange traits, namely *E*, *A*_max_, and *g*_s_, expressed statistically significant variation (Table 2, Fig 1). Specifically, across cultivars both Cabernet sauvignon cultivars (CS29, CS412) exhibited among the highest physiological trait values, with *E* rates of 3.5 ± 0.01 and 3.1 ± 0.01 mmol H_2_O m^-2^ s^-1^, respectively; *A*_max_ values of 24.2 ± 0.8 and 24.3 ± 0.8 *μ*mol CO_2_ m^-2^ s^-1^, respectively; and *g*_s_ values of 0.34 ± 0.02 and 0.37 ± 0.01 mol m^-2^ s^-1^, respectively, highlighting that these clones generally express traits consistent with high rates of leaf-level resource aquistion. Other cultivars exhibiting high leaf-level resource acquisition, denoted by high values of include Riesling cultivars (R171 and R23) and Sauvignon blanc (SB906). Additionally, Merlot cultivars (M181 and M348), exhibited lower resource acquisition associated with reduced rates of *E* (2.90 ± 0.16 and 2.67 ± 0.12 mmol H_2_O m^-2^ s^-1^, respectively), and *A*_max_ (20.0 ± 0.98 and 19.2 ± 1.07: *μ*mol CO_2_ m^-2^ s^-1^, respectively).

**Table 2:** Mean trait values measured across 12 wine grape cultivars with ± standard error (*n* ≈ 15 per cultivar).

		Physiological			Chemical		Water relations				Morphological		
Variety	Cultivars	*E*	*A*_max_	*g*_s_	N	C	δ ^13^C	Ψ_pd_	WUE_inst_	WUE_intr_	Area	LDMC	LMA
**Cabernet franc**	CF314	1.85	22.54	0.22	2.82	46.75	−28.26	5.94	12.47	106.34	151.57	255.67	67.04
		±0.080	±0.66	±0.01	±0.12	±0.15	±0.32	±0.52	±0.65	±5.30	±5.71	±5.57	±1.47
	CF327	1.29	23.52	0.2	2.72	45.39	−27.75	5.53	19.28	122.15	140.28	271.88	75.76
		±0.0	±0.87	±0.01	±0.08	±0.15	±0.22	±0.23	±1.39	±7.93	±7.37	±4.66	±1.66
**Cabernet sauvignon**	CS29	3.49	24.23	0.34	2.8	45.25	−28.44	7.06	7.03	73.75	137.72	286.79	73.19
		±0.089	±0.75	±0.02	±0.11	±0.16	±0.23	±0.45	±0.30	±4.76	±5.8	±23.82	±1.43
	CS412	3.09	24.3	0.37	2.66	45.27	−28.21	6.89	7.88	66.17	135.17	280.46	73.96
		±0.011	±0.75	±0.01	±0.12	±0.16	±0.24	±0.43	±0.24	±2.52	±4.46	±14.20	±2.98
Merlot	M181	2.90	20	0.2	2.96	45.93	−27.29	6.96	7.07	104.94	180.8	267.99	71.11
		±0.16	±0.98	±0.02	±0.08	±0.28	±0.21	±0.27	±0.35	±6.67	±8.61	±3.52	±3.27
	M348	2.67	19.2	0.18	2.92	45.94	−28.11	6.28	7.17	109.37	167.91	265.7	70.13
		±0.12	±1.07	±0.01	±0.07	±0.07	±0.21	±0.36	±0.20	±2.94	±9.88	±3.31	±1.37
Pinot noir	PN828	4.38	21.47	0.26	2.85	46.06	−28.02	5.69	5.00	84.22	134.99	280.13	77.32
		±0.20	±0.67	±0.01	±0.11	±0.14	±0.34	±0.33	±0.22	±3.70	±13.24	±15.76	±3.71
	PN89	3.93	21.01	0.28	2.78	46.13	−27.8	6.03	5.43	78.25	155.56	247.67	71.05
		±0.20	±0.85	±0.02	±0.11	±0.18	±0.20	±0.2	±0.18	±3.62	±8.98	±5.35	±2.55
Riesling	R171	3.93	19.75	0.26	2.41	45.61	−28.43	3.95	5.64	78.09	169.79	254.64	69.07
		±0.13	±0.77	±0.01	±0.06	±0.16	±0.33	±0.19	±0.17	±3.17	±11.02	±4.93	±2.59
	R23	3.52	21.08	0.28	2.67	46.65	−26.83	4.32	5.18	80.50	157.56	248.67	73.06
		±0.20	±0.51	±0.02	±0.05	±0.15	±0.55	±0.13	±0.31	±6.14	±9.4	±4.75	±1.69
Sauvignon blanc	SB906	4.21	22.29	0.32	2.62	45.7	−28.12	6.03	4.73	71.12	154.9	229.56	66.48
		±0.16	±0.79	±0.01	±0.07	±0.17	±0.41	±0.18	±0.24	±3.96	±9.53	±4.91	±1.42
Viognier	V642	4.80	22.61	0.18	2.78	45.26	−27.43	6.08	8.41	125.90	121.19	258.53	72.29
		±0.081	±0.47	±0.01	±0.05	±0.12	±0.27	±0.19	±0.11	±3.38	±5.29	±18.47	±4.12

*E*: mmol H_2_O m^-2^ s^-1^; *A*_max_: *μ*mol CO_2_ m^-2^ s^-1^; *g*_s_: mol m^-2^ s^-1^; WUE_inst_: *μ*mol CO_2_ mmol^-1^ H_2_O; WUE_intr_: *μ*mol CO_2_ mol^-1^ H_2_O; N: % dry mass; C: % dry mass; δ^13^ C: ‰; Ψ_pd_: MPa; Area: cm^2^; LDMC: mg g^-1^; LMA: g m^-2^.

Chemical traits associated with leaf construction cost (leaf C), resource acquisition (leaf N), and plant-water relations (δ ^13^C) all varied little across cultivars (Fig 1). Here, leaf C exhibited the least variation (CV = 1.7) with cross-cultivar ranges of 47.5–42.6%, and 75% of the data fell between 44.9–46.7% dry mass. Riesling and Cabernet franc cultivars exhibited the highest leaf C concentrations, with values ranging from 45.6–46.8% dry mass. Leaf N exhibited higher variation (CV = 12.7), ranging from 1.6–3.6%, with Merlot cultivars exhibiting the highest mean leaf N concentrations (2.9%), compared to Riesling (cultivar 171), which had the lowest mean concentration of leaf N of 2.4% dry mass (Table 2). Additionally, for δ ^13^C, most cultivar mean values clustered around −28‰, with Riesling clone 23 presenting the lowest average (−26.83 ± 0.55‰), suggesting potentially lower long-term enhanced WUE. Compared to other traits except for leaf C values, δ ^13^C exhibited the most limited variation (CV = 4.4).

Morphological traits, including leaf area, LDMC, and LMA, exhibited greater variation among cultivars. Specifically, both Merlot cultivars (M181, M348) expressed on average the largest leaf area (167.91 ± 9.88 to 180.8 ± 8.61 cm^2^), while the Viognier cultivar (V642) and one Pinot noir cultivar (PN828) expressed the smallest average leaf areas (121.2 ± 5.3 and 134.99 ± 13.2 cm^2^, respectively; Table 2). Traits associated with leaf construction, namely LDMC, was the highest in the Cabernet sauvignon (CS29 and CS412) cultivars (286.79 ± 23.82 and 280.46 ± 14.20 mg g^-1^, respectively), with LMA following a similar trend, being highest in the CS cultivars along with PN828 (range = 73.2 ± 1.4 to 77.3 ± 3.7 g m^-2^).

### 3.2 Reflectance spectra and PLSR models

**Fig 2:**
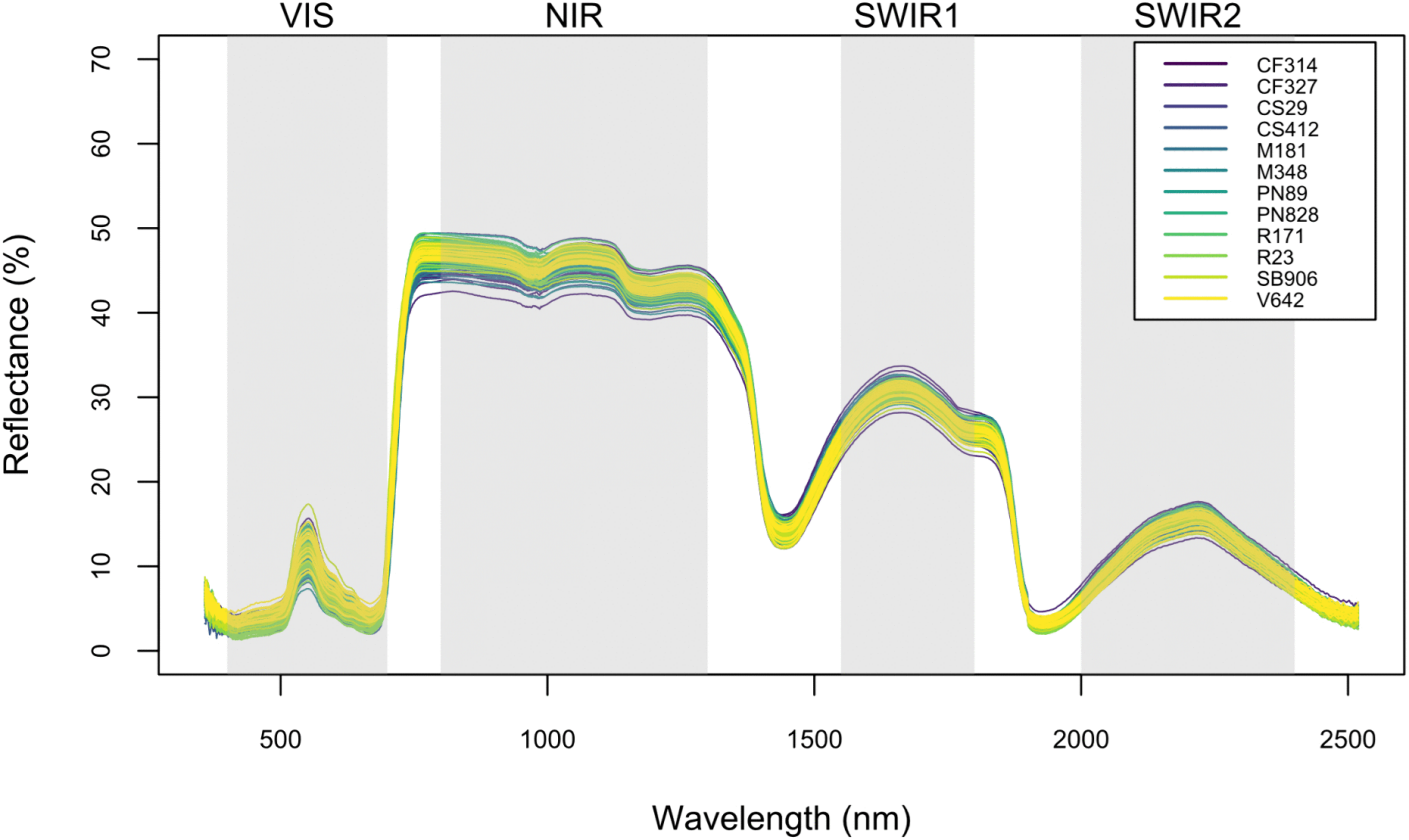
Spectra reflectance quantiles for 12 wine grape cultivars. Key wavebands are highlighted by the grey bars. VIS: visible light spectrum (380–780 nm); NIR: near-infrared (780–1400 nm); SWIR1: shortwave infrared radiation 1 (1550–1750 nm); SWIR2: shortwave infrared radiation 2 (2000–2300 nm).

**Fig 3:**
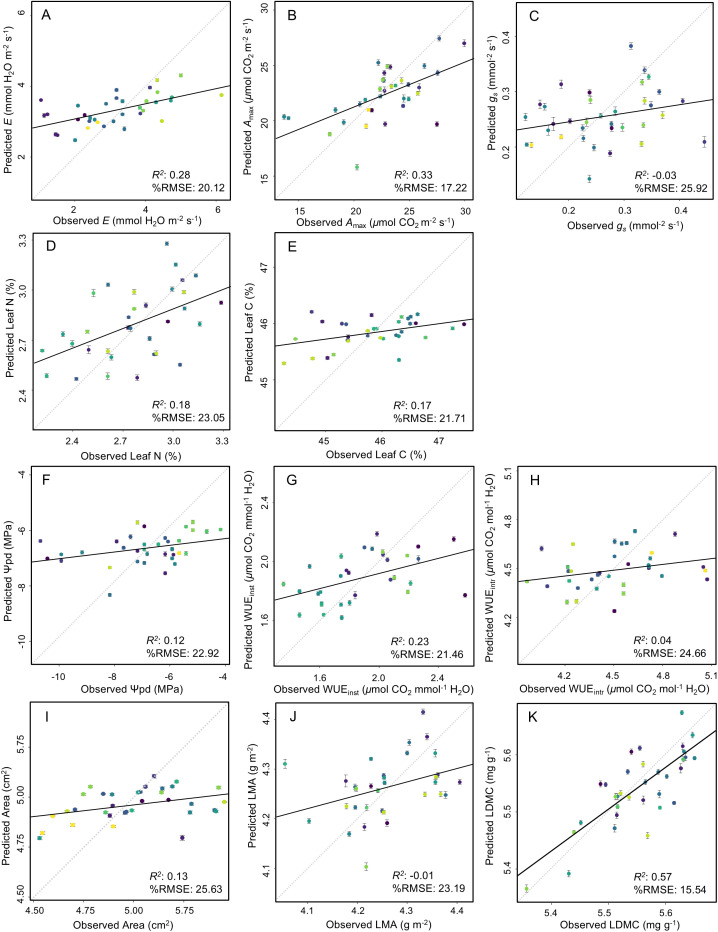
Results of partial least squares regression (PLSR) models predicting 11 leaf functional traits in 12 wine grape cultivars. Panels show the data points used to validate the models fitted to a set of calibration data, based on a fully randomized split (*n*_cal_ and *n*_val_ presented in Table 3). The black lines represent linear regression model fits between the observed and predicted traits, and the dotted grey lines present a 1:1 relationship. The bottom-right corner of each plot shows both the *R*^2^ and the %RMSE for each model. Plots G–K present log-transformed trait data as shown in Table 3.

The spectra quantiles for all 12 wine grape cultivars (total *n* = 178 leaves) indicated that, with the exception of two leaves (both removed due to measurement errors), leaves selected for our analysis here were free of major damage or underlying structural issues that might compromise leaf health (Fig 2). As presented in Table 3 and visualised in Fig 3, PLSR models parameterized under random data splits yielded, on average, more statistically significant components, which in turn supported PLSR models with stronger predictive capacity. Specifically, of the 12 traits analyzed in our PLSR framework, models fitted on fully randomized datasets were stronger (vs. those balanced across cultivars) in 7 of 11 instances (with one instance of PLSR models not converging).

**Table 3:** Partial least squares regression model fits predicting leaf trait variation across 12 wine grape cultivars. Different modelling frameworks denoted in the “Data Split” column, refer to manner in which data was divided into calibration (80%) and validation (20%) datasets for model fitting, where results from the “Cultivar” data split entail calibration/ validation datasets with approximately equal proportions of observations from all 12 wine grape cultivars, and the “Random” approach selected data for the calibration and validation datasets randomly.

Data Split	Trait group	Trait	*n*_obs_	*n*_cal_	*n*_val_	*n*_comp_	RMSE	*R* ^2^	%RMSE
Cultivar	Physiological	*E*	173	137	36	3	1.04	0.19	19.77
		*g*_s_	173	137	36	2	0.08	−0.02	26.67
	Chemical	N	163	127	36	3	0.28	0.35	21.19
		C	162	126	36	1	0.71	0.14	25.38
	Water relations	Ψ_pd_	172	136	36	4	1.55	−0.02	21.36
		log-WUE_inst_	160	126	34	4	0.30	0.02	26.31
		log-WUE_intr_	172	136	36	3	0.29	0.09	22.91
	Morphological	log-Area	168	132	36	1	0.19	0.26	20.11
		log-LDMC	161	125	36	9	0.06	0.35	20.38
		log-LMA	161	125	36	5	5.54	0.29	16.18
Random	Physiological	*E*	173	138	35	2	1.03	0.28	20.12
		*A*_max_	173	138	35	9	2.80	0.33	17.22
		*g*_s_	173	138	35	8	0.08	−0.03	25.92
	Chemical	N	163	130	33	3	0.25	0.18	23.05
		C	162	129	33	2	0.69	0.17	21.71
	Water relations	Ψ_pd_	172	137	35	3	1.49	0.12	22.92
		log-WUE_intr_	173	138	35	4	0.27	0.04	24.66
		log-WUE_inst_	160	128	32	4	0.26	0.23	21.46
	Morphological	log-Area	168	134	34	2	0.24	0.13	25.63
		log-LDMC	161	128	33	11	0.05	0.57	15.54
		log-LMA	161	128	33	5	0.08	−0.01	23.19

*n*_obs_: total number of observations; *n*_val_: total number of validation observations; *n*_comp_: number of components; RSME: root mean square error; RSME%: percent root mean square error (validation data); *E*: mmol H_2_O m^-2^ s^-1^; *A*_max_: *μ*mol CO_2_ m^-2^ s^-1^; *g*_s_: mol m^-2^ s^-1^; WUE_inst_: *μ*mol CO_2_ mmol^-1^ H_2_O; WUE_intr_: *μ*mol CO_2_ mol ⁻ ¹ H_2_O; N: % dry mass; C: % dry mass; Ψ_pd_: MPa; Area: cm^2^; LDMC: mg g^-1^; LMA: g m^-2^. Cultivar split did not successfully model *A*_max_, nor did either data-splitting approach model δ ^13^C (there were no latent component that explained the covariance).

Specifically, our PLSR models indicated that the traits most well predicted through models trained on fully randomized calibration and validation datasets were LDMC (*R*^2^ = 0.57, %RMSE = 15.54), *A*_max_ (*R*^2^ = 0.33, %RMSE = 17.22), and *E* (*R*^2^ = 0.28, %RMSE = 20.12) (Fig 3). PLSR models with moderate-to-low *R*^2^ included those fitted for leaf C and N (*R*^2^ = 0.17, %RMSE = 21.71, and *R*^2^ = 0.18, %RMSE = 23.05, respectively), Ψ_pd_ (*R*^2^ = 0.12, %RMSE = 22.92), and log-Area (*R*^2^ = 0.13, %RMSE = 25.63). Comparatively, PLSR models based on fully randomized data splits were least successful in predicting *g*_s_ and WUE_intr_, as indicated by near-zero *R*^2^ values. Moreover, models with the strongest fits under a randomized data-splitting structure (namely, LDMC, *A*_max_, and *E*) tended to be traits with high variability (as per Tables 1 and 2), and those functionally interconnected through their roles in carbon assimilation and water use. The PLSR model was not effective in predicting δ ^13^C due to the reduced variability exhibited within-cultivars, and regardless of the data-splitting approach or alternative component selection methods, there was an optimal component output of 0, indicating that there is no latent component that explains the covariance between our predictor and response variables [46].

When calibration and validation datasets contained equal proportions of data from each cultivar, PLSR models predicting leaf morphological traits (log-Area, log-LMA) expressed stronger predictive capacity with *R*^2^ values of 0.26 (%RMSE = 20.11) and 0.29 (%RMSE = 16.18), respectively. This was also the case for a PLSR model predicting leaf N (*R*^2^ = 0.35, %RMSE = 21.19). This likely owes to leaf morphological traits varying more widely among cultivars as they are strongly regulated by leaf genetics (for leaf shape and overall size), with environmental factors influencing their plasticity [47]. However, PLSR models based on balanced calibration and validation datasets across cultivars were not stronger for *E*, *A*_max_, leaf C, Ψ_pd_, WUE_inst_, and LDMC. Instead, these traits are more robustly predicted by PLSR models when the training and validation datasets were generated using a randomized data split (*R*^2^ = 0.28, 0.33, 0.17, 0.23, and 0.57, respectively). When comparing model performance across the CVs for each trait, there was a strong inverse correlation with the Cultivar data-splitting approach and a weaker inverse correlation with the Random data-splitting approach. To confirm the influence of our data splitting approaches, we performed a Peason correlated test between prediction *R*^2^ values and the degree of variation for each trait (CV). For both data splitting approaches, there were negative *r* values, with −0.55 for Cultivar splits, and −0.22 for the Random split, meaning that as CV increases, *R*^2^ decreases more strongly for Cultivar splits, which is evident in the more plastic physiological traits.

**Fig 4:**
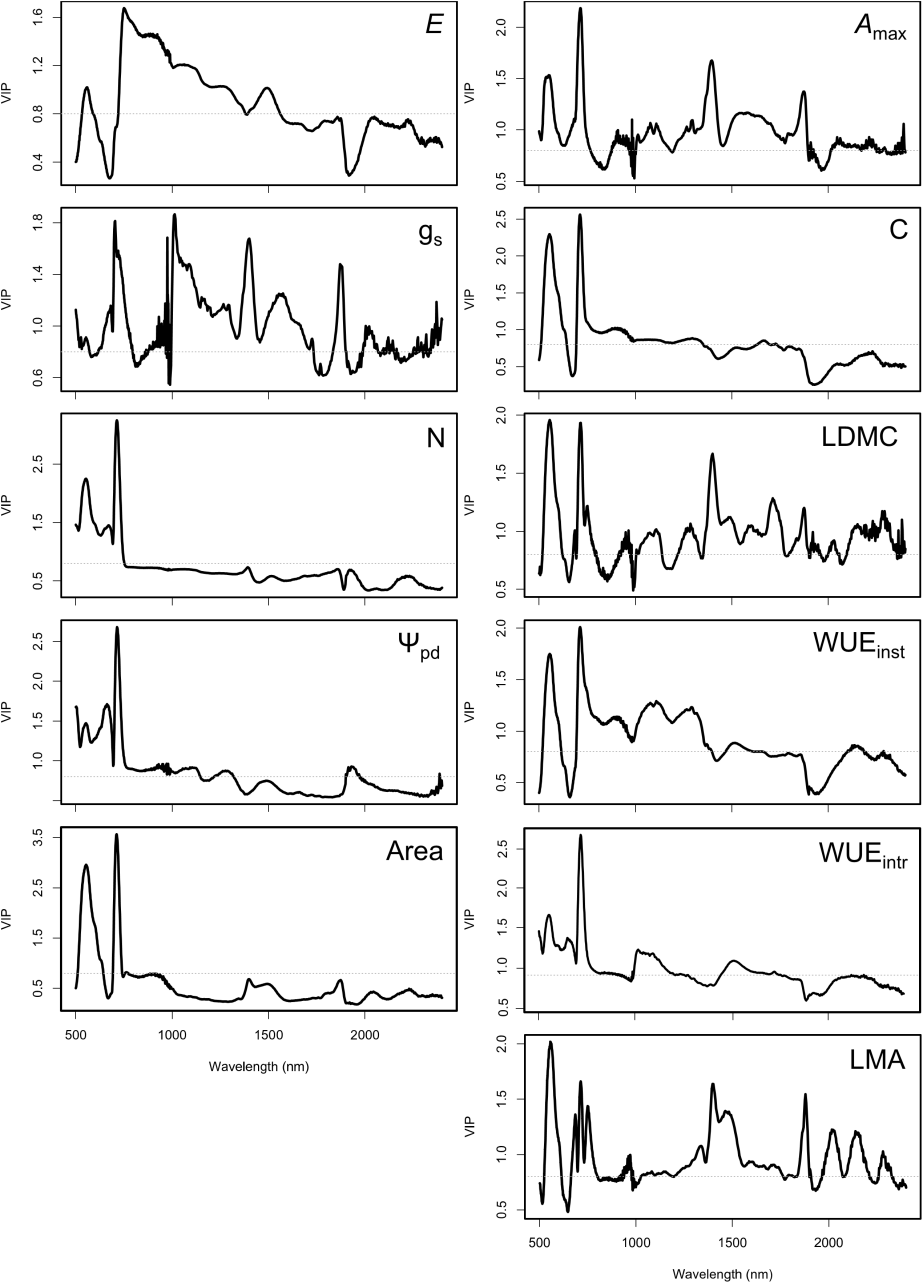
Variable importance in projection (VIP) scores associated with partial least squares regression (PLSR) models for calibration data using a “random” split. The dashed line marks the VIP score threshold of 0.8, indicating the cutoff for identifying the most important wavebands for trait predictions; values ≥ 1 highlight more influential variables.

Based on the VIP scores dervived from our PLSR modelling procedure for traits with moderate *R*^2^ with randomized data splits, our analysis highlighted the strong influence of wave bands in the the red-edge (680−750 nm) and near-infrared region (780−1400 nm) in predicting physiological traits including *E*, *A*_max_, as well as for two water relation traits (Ψ_pd_ and WUE_inst_), where VIP scores were > 1, and in multiple instances approaching 2 (Fig 4). We also detected a strong influence of wave bands in the visible light region (400−750 nm) in predicting leaf C concentrations, and LDMC (VIP scores > 1.5), with wavebands in the short-wave infrared-2 region (2,000−2,290 nm) also having a strong influence on PLSR models predicting LDMC and LMA (VIP > 1.3). However, VIPs associated with PLSR models predicting leaf N concentrations did not follow the expected patterns, namely VIP < 1 in the SWIR1 region (1,550−1,750 nm), potentially due to the limited variability in our leaf N data, leading to reduced ability to capture trait variation commonly associated with reflectance in this region. While the predictive power of our PLSR models depended on the data-splitting procedure and parameterization, VIP scores were not sensitive to the configuration of this particular model-fitting procedure.

## 4 Discussion

This study contributes to the growing body of research demonstrating the application of reflectance spectroscopy and PLSR modelling for quantifying trait variation that exists among crop cultivars, and within plant species more broadly. Previous work has established this approach to trait estimation across different species [25,26,42], with more recent studies exploring its application in crops, including wine grapes. Specifically, our previous work focused on predicting five leaf traits associated with aboveground resource capture (e.g., photosynthetic carbon assimilation, RuBisCO carboxylation, and electron transport) across varieties, but not cultivars of the same variety [30]. Here, we contribute new findings that traits related to leaf-level physiology (*E*, *A*_max_, *g*_s_), plant-water relations (Ψ_pd_, δ ^13^C, WUE_intr_, WUE_inst_), stoichiometry (leaf C and N), and leaf construction (leaf area, LMA, LDMC) vary across cultivars within varieties (Fig 1, Tables 1 and 2), and that reflectance spectroscopy can indeed predict this finer-scale trait variation (Fig 3, Table 3). The importance of understanding and quantifying such resolved trait variation as a driver for plant- and ecosystem-level resilience [18,48] has gained increased attention, motivating an interest in using reflectance spectroscopy and PLSR modelling for functional trait characterisation, especially in agroecosystems and crop breeding programs [25]. Here, we explored the extent and sources of functional trait variation across a diverse genetic gradient of wine grape cultivars using both field-based research methods and leaf-level spectral data.

Our findings demonstrate that plant traits varied across cultivars, indicating a strong influence of wine grape genotypes on phenotypic trait expression. This variation was most evident in leaf physiological (E, *A*_max_, *g*_s_) and water-use traits (WUE_inst_, WUE_intr_), with less pronounced variation in leaf chemical and morphological traits, specifically in leaf C and N concentrations, LMA, and LDMC. Two water relations traits, namely Ψ_pd_ and δ ^13^C, also exhibited limited variation across cultivars. Similar patterns of more pronounced variability in gas exchange-based physiological traits and water-use traits, vs. more constrained variation in structural and chemical traits, have been documented in other single-species studies [49–52].

Also in line with previous studies on wine grape varieties and other plant species [24,30,39,53,54], we found that the red-edge spectral bands in the ~ 680–750 nm range exert the strongest influence on trait estimation for a majority of traits measured here, including *A*_max_, leaf C and N, Ψ_pd_, WUE_inst_, LDMC, and leaf area. In all of these cases, VIP scores for red-edge were > 2, such that the red-edge region clearly emerged as one of the more important wavebands for PLSR modelling [24,55]. Although WUE_intr_ and WUE_inst_ are not directly optical traits, they are indicative of stomatal relations and therefore covary with other resource-use traits captured by red-edge bands [38]. These findings indicate that reflectance spectroscopy has the potential to capture subtle and cultivar-specific spectral signatures related to stress responses, particularly in traits with strong optical properties (i.e., physiological, stoichiometric, and structural traits). However, VIP scores are sensitive to model performance, meaning their interpretation is viable and biologically-meaningful only if PLSR model fits were “successful” [42].

Consistent with previous studies as well, we find that reflectance profiles in the lower SWIR range wavebands (i.e., SWIR 1 in the 1550–1750 nm range) is critically important in predicting physiological (*A*_max_) and water-use traits, as well as LDMC. The SWIR 1 region contains strong water absorption features, making it highly sensitive to variation in leaf water content [31]. Reflectance values in this region also provide an indirect signal to water-use traits since leaf water status is strongly associated with leaf physiological processes [39,40]. The ability to detect water-stress traits in wine grapes is an essential advancement in applications of reflectance spectroscopy towards crop ecophysiology research and practice, since traditional methods for water-stress analysis remain time-consuming. Detecting water status through proximal sensing, like reflectance spectroscopy, can also ensure closely related cultivars with differing water statuses receive more targeted watering regimes.

In our analysis, we found that PLSR models were most effective in predicting three physiological and morphological leaf traits with a predictive power ranging from 28% to 52%, namely *E*, *A*_max_, and LDMC. Additionally, our PLSR models were successful, albeit more moderately, in predicting leaf C and N concentrations, Ψ_pd_, and leaf area. Compared with other single-species studies [24,30,31] our PLSR models were less precise in their trait predictions. For example, in similar studies, the predictive power of the PLSR models for leaf physiological traits based on gas exchange across only six wine grape varieties was much higher (i.e., 18–62%) [30]. Our findings here highlight broader potential to associated functional traits and physiological status with reflectance spectra signatures, potentially addressing a limitation to the degree of variation that reflectance spectroscopy can estimate. However, in multi-cultivar agroecosystems like vineyards, where multiple cultivars often coexist in a single environment, our results suggest reflectance spectroscopy is a useful tool for detecting certain cultivar-specific stress responses.

One potential explanation for the lower predictive power observed in this study is the reduced variability of several leaf traits across our entire dataset, at the cultivar level. Compared to our previous work [30] the magnitude of trait variation for certain traits was lower in our study here. For example, the CVs for leaf N and LMA in previous studies on wine grapes with fewer cultivars were 13.7% and 12.8%, respectively [30], as compared to CV values of 12.6% and 9.6% for these same traits observed here. One expectation might be that this reduced trait variability reduces PLSR model performance, while the relative trends in predictive power among the different data-splitting approaches remained consistent. In contrast, comparing variability in *A*_max_ between studies%, CV values were nearly identical (~34.8). Accordingly, the predictive performance of PLSR models was nearly identical between both our and comparable studies [30].

The predictive power of PLSR models relies on the ability of models to identify covariance structures between predictor variables and response traits. Therefore, if trait variation is more limited (i.e., a lower CV), one might expect the strength of covariance patterns to be weaker, leading to less clear/weaker trait predictions [26]. The patterns found in our study generally support the interpretation that PLSR model performance is influenced by the magnitude of trait variation within a given dataset. However, model performance is not solely constrained by this relationship, and less direct spectral-trait interactions more strongly influence model performance [56].

By extension, the ability of PLSR models to predict traits across wine grape cultivars depended on data-splitting parameterization. Fully randomizing calibration and validation datasets generally led to higher explanatory power (*R*^2^ and %RMSE values) across 7 of the 11 traits successfully modelled here. When trait variation across a dataset is higher, non-random data splits may not capture the entirety of trait variation, and in turn provide less reliable performance metrics. More specifically, traits that are A) highly plastic across environmental conditions and B) less constrained within genotypes (or other factors)—such as *A*_max_—may be more accurately modelled using a randomized split. This is because a randomized approach would be expected to better capture the entire breadth of trait values, whereas a targeted data split (i.e., balanced across genotypes) is more likely to miss the entire extent of trait distributions. Under this scenario, PLSR models fitted to validation data may be less strong in their predictive ability [56–58]. Thus, our results suggest that ahead of model calibration, data splits need to be assessed based on the general variability of the training data and how this variation is structured [26,42]. Data-splitting strategies can also yield different outcomes depending on the trait being assessed; traits with more pronounced genetic specificity may be more accurately modelled using a cultivar split (i.e., area).

## 5 Conclusions

Our study contributes new data demonstrating that functional traits vary significantly among and within across wine grape cultivars, with cultivars of the same variety expressing considerable variation in leaf traits related to physiology, plant water relations, leaf tissue chemistry, and leaf morphology. Physiological and water-use traits are particularly variable among closely related cultivars, highlighting their potential use for selecting resilient cultivars for breeding programs and climate adaptation strategies. We also contribute among the first set of results demonstrating that reflectance spectroscopy can detect and estimate this fine-scale intraspecific trait variation, which implies strong potential to scale non-destructive trait monitoring from individual plants to large-scale agricultural systems. However, our results also show that predictive models are sensitive to model parameterization—specifically decisions surrounding calibration and validation dataset construction—in a manner that is associated with trait variation: reflectance-based trait models generally perform better when wider trait variation is present in both training and validation data. Taken together, our analysis highlights the variation in functional traits across genetic gradients and contributes to the growing body of research exploring reflectance-based approaches to quantify intraspecific variation in ecophysiological traits of plants and crops [14,18,30,59].

## Abbreviations

*A*_max_: Maximum photosynthetic rate
C: Carbon
CF314: Cabernet Franc 314
CF327: Cabernet Franc 327
CS29: Cabernet Sauvignon 29
CS412: Cabernet Sauvignon 412
CV: Coefficient of variation
*E*: Transpiration
*g*_s_: Stomatal conductance
HTP: High-throughput phenotyping
LDMC: Leaf dry matter content
LMA: Leaf mass per area
M181: Merlot 181
M348: Merlot 348
N: Nitrogen
PLSR: Partial least squares regression model
PN828: Pinot Noir 828
PN89: Pinot Noir 89
R171: Riesling 171
R23: Riesling 23
RSME: Root mean square error
SB908: Sauvignon blanc 906
V642: Viognier 642
VIP: Variable importance in projection
WUE_intr_: Intrinsic water-use efficiency
WUE_inst_: Instantaneous water-use efficiency
δ ^13^C: Stable isotopic carbon fraction
Ψ_pd_: Predawn water potential
